# Quality of Life and Job Satisfaction of Dispensing Pharmacists Practicing in Tehran Private-sector Pharmacies 

**Published:** 2012

**Authors:** Marzieh Majd, Farshad Hashemian, Farnaz Younesi Sisi, Masoud Jalal, Zahra Majd

**Affiliations:** a *Department of Clinical Pharmacy, Pharmaceutical Sciences Branch, Islamic Azad University (IAU), Tehran, Iran.*; b *Department of Chemistry, Pharmaceutical Sciences Branch, Islamic Azad University (IAU), Tehran, Iran. *; c *Faculty of Pharmacy, Tehran University of Medical Sciences, Tehran, Iran. *

**Keywords:** Job satisfaction, Dispensing pharmacists, Quality of life, General health, Tehran

## Abstract

As there is no evidence of previous studies on evaluating the level of job satisfaction and the major causes of dissatisfaction among the pharmacists in Iran, this study was designed.

This study is a cross-sectional descriptive analysis of pharmacists practicing in Tehran private-sector pharmacies. We selected a stratified random sampling using number of prescriptions as a variable for stratification. The questionnaire was divided into three sections containing the demographic characteristics, general health perception and job satisfaction.

Of all the participants, 62% were the owners of pharmacies and 38% were pharmacists in charge (non-owner). Seventy-eight percent of respondents reported satisfaction about their psychological and physical state. Just 11% of pharmacists were financially satisfied and 49% felt relaxed at the workplace. There was no correlation between the satisfaction and owning the pharmacy or sex of respondents. Spearman›s correlation showed that the income satisfaction correlated negatively with age (p ≤ 0.001) and years of experience (p < 0.05). Moreover, the average working hours was significantly higher among men compared to women (p < 0.01) and among owners relative to non-owners (p < 0.05).

Overall, general health perception and quality of life among the respondents were at satisfactory level. However, work-related satisfaction was not high enough and most interviewed pharmacists were financially dissatisfied.

## Introduction

According to the HRSA (The Health Resources and Services Administration) report, pharmacist population in the USA has been increased dramatically from 196000 in the year 2000 to near 225000 in 2010. This growth is due to the increased demand related to some alteration of social needs to pharmacist services like growing the elderly population (1). Currently, pharmacy practice includes more modern services in order to optimize health outcomes for patients. With regard to the importance of pharmacists’ role and quality of services on the health care system, several studies have examined the key factors threatening pharmacy profession. 

In 1988, Barnett and Kimberlin mentioned that the job satisfaction among pharmacists promotes their performance and consequently have a positive impact on patients (2). Many studies have attempted to determine the factors affecting pharmacists’ job satisfaction. Mott *et al. *conducted the study on pharmacists’ attitudes towards work life. They noted that 67.2% of pharmacists were satisfied with their job, although they reported job stress, excessive work load and work-home conflict (3).

Furthermore, it is investigated that the level of job satisfaction among pharmacists is influenced by types of their practice setting (4-8). One study in 2004 indicated that most pharmacists were satisfied with their positions, although those practicing in the chain pharmacies reported much lower levels of satisfaction compared to other settings (8). The results were similar to those reported by others (6). Job stress among pharmacists has been reported by several studies (4, 5, 6, 9, 10). Moreover, it is reported that dispensing pharmacists experienced more job stress in comparison with the consultant pharmacists. As a result, they are more likely to experience job dissatisfaction than their peers in other settings (4). Another study in 2004 investigated that the workday’s responsibilities of pharmacists might not be proportionate to the qualifications they have gained during their education (11). Thus, it can be one of the contributing factors of their job dissatisfaction.

Like other countries, pharmacists in Iran are facing a number of challenging issues. Pharmacists who work in pharmacies frequently express job dissatisfaction in the annual meetings. However, there is no evidence of previous studies on evaluating the level of job satisfaction and the leading causes of dissatisfaction among dispensing pharmacists in Iran.

Ultimately, the present study was designed in order to evaluate the level of job satisfaction and quality of life among dispensing pharmacists practicing in Tehran private-sector pharmacies.

## Experimental

*Methods*

This study is a cross-sectional descriptive analysis of pharmacists practicing in Tehran private-sector pharmacies which was conducted in Tehran, Iran.

A sample size of 110 private pharmacies was determined according to Bartlett and colleagues determining sample size research (12). Since the number of prescriptions can be a predictor of pharmacy income, a stratified random sampling was selected based on the number of prescriptions submitted by insurance companies as a variable for the stratification of pharmacies.

Questionnaires were distributed among pharmacists who are working in the selected pharmacies as either an owner or non-owner (in charge of responsibility), by trained questioners.

The questionnaire was validated under supervision of Iranian Pharmacists Association.

It was designed by three types of questions: I: Demographic characteristics, including: Age, sex, years of experiences and level of education; II: General health perception: We considered the following variables as predictors of their general health perception: Physical and psychological health state; Quality of life level; Time spent with family. III. Job satisfaction: In this part, respondents were asked to answer questions related to their income satisfaction, the perception of being respected by patients, the mental function while working in pharmacy, the pharmacist-physician relationship quality, and also the average hours worked per day.

In order to evaluate the level of job satisfaction and quality of life, the questions were designed based on 5-point scale template. For the simplification of analysis, we divided answers into two categories according to the type of questions. Response values of 4 and 5 were reported as “satisfactory level”.

Statistical analysis was done by SPSS software. P-values ≤ 0.05 were considered statistically significant.

This study was approved by “Ethics Committee” of Islamic Azad University of Pharmaceutical Sciences Branch (No: 14250).

## Results

*Demographic characteristics*

Table 1 gives information about respondents› demographics. Sixty-four percent of participants were men. The age range was between 24 and 71 years with the mean age of 42. The minimum year in practice was 1 and maximum was 52, the mean year in practice was 15.

Nearly half of the respondents (49%) worked more than 10 h daily. Of all the participants, 62% were the owners of pharmacies and 38% were in charge of responsibility (non-owner).

**Table 1:** Demographic characteristics

**Characteristics of pharmacists**	(%)
**Gender**	
F	(36%)
M	(64%)
Age	
≤ 30	(20%)
31-45	(42%)
46-60	(28%)
≥ 61	(5%)
Missing	(4%)
**Responsibility in pharmacy**	
Owner	(62%)
Non-owner	(38%)
**Years in practice**	
≤ 5	(19%)
6-20	(54%)
21-35	(19%)
≥ 35	(5%)
Missing	(2%)
**Average hours worked per day**	
≤ 4-5	(11%)
8-10	(40%)
> 10	(49%)
**Degree**	
PhD	(2%)
Pharm. D	(98%)

*General health perception*

Table 2 illustrates the proportion of respondents rating their general health level “satisfactory”. Seventy-eight percent of respondents reported satisfaction about their psychological and physical state. Sixty-five percent reported satisfactory quality of life during the last month.

**Table 2:** General health perception.

**Variable**	**Answer**
• At your age, in general, how would you rate/describe your health? (physically and psychologically) • How would you rate your overall quality of life during the last month? • How would you rate your overall quality of life compared to 10 years ago?	"**satisfactory**"
	(78%) (65%) (52%)
• Average time spent with family per day (h): < 1 1-2 2-4 ≥ 4	(21%) (31%) (29%) (19%)

*Job satisfaction*

In Table 3, we summarized various factors as predictors of profession satisfaction. Just 11% of pharmacists were financially satisfied. Of all the interviewed pharmacists, fifty percent reported that they got due respect from patients and 67 percent stated satisfactory relationships with physicians. In the questions addressed mental function while working in pharmacy, 74%, 69% and 67% of respondents rated concentrated, mood and energetic respectively, at “satisfactory level”. Forty-nine percent felt relaxed at the workplace.

Respondents were also asked whether they are proud to work as a pharmacist or not, and 73% answered “agree” to this question.

**Table 3:** Factors affecting work-related satisfaction

**Variable**	**Answer**
Income/financial satisfaction Getting due respect from patients Pharmacist-physician relationship quality	**"satisfactory level"**
	(11%) (50%) (67%)
• Mental function: How would you describe/rate the following when you are at pharmacy? Concentrated Mood Energetic	**"satisfactory level"**
	(74%) (69%) (67%)
Feel relaxed while working at pharmacy	(49%)
I am proud to work as a pharmacist.	**Agree** (73%)

*Factors affecting the dependent variables*

A chi-square analysis (cross-tabulation) showed no significant differences in physical and psychological status, quality of life level, income satisfaction, job stress and other practice related variables according to the gender and type of responsibility (owner or non-owner). Moreover, the average of working hours was significantly higher among men compared to women (p < 0.01) and among owners compared to non-owners (p < 0.05) (Table 4).

**Table 4:** Factors affecting the dependent variables

**Variable**	**Owner**	**Non-owner**	**p-value**	**Male**	**Female**	**p-value**
**Physically and psychologically healthy**	(73%)	(85%)	NS	(74%)	(84%)	NS
**Quality of life satisfaction**	(60%)	(73%)	NS	(62%)	(72%)	NS
**Income satisfaction**	(11%)	(12%)	NS	(12%)	(9%)	NS
**Mental status while working:** **• Energetic** **• Mood** **• Concentrated** **• Feel relaxed**	(66%) (66%) (73%) (45%)	(68%) (73%) (76%) (56%)	NS	(72%) (72%) (74%) (47%)	(56%) (63%) (75%) (53%)	NS
**Average hours worked per day:** **≤ 4-5** **8-10** **> 10**	(5%) (35%) (60%)	(21%) (47%) (32%)	< 0.05a	(9%) (29%) (62%)	(16%) (59%) (25%)	< 0.01a
**Average time spent with family per day:** **≤ 2 h** **2-4 h** **> 4 h**	(57%) (27%) (16%)	(44%) (32%) (24%)	NS	(62%) (22%) (16%)	(34%) (41%) (25%)	NS
**Proud to work as a pharmacist**	(73%)	(74%)	NS	(78%)	(66%)	NS

Spearman’s non-parametric correlation revealed that the income satisfaction correlated negatively with age (p ≤ 0.001) and years of experience (p < 0.05) (Table 5). Other work-related variables were not significantly correlated with age and work experience. 

**Table 5:** Factors correlated negatively with financial satisfaction

**Variable**	**Spearman's rho**	**p-value**
**Age**	-0.349	0.001
**Years in practice**	-0.271	0.011a

a Spearman’s correlation is significant at 0.05 level.

## Discussion

While most of pharmacy graduates in Iran employ as a dispensing pharmacists, our study focused on evaluating the job satisfaction and quality of life among dispensing pharmacists.

Several studies have proved that job satisfaction is dramatically influenced by the pharmacists practice settings. Chain pharmacists have been found to experience a lower extent of job satisfaction relative to others (5, 6, 7, 13). In study conducted among the employees of long-term care pharmacy team, dispensing pharmacists and pharmacy technicians reported 

lower levels of job satisfaction compared to consultant pharmacists (4).

Our findings showed that the general health and quality of life among respondents were at satisfactory level. In this study, the largest percentage of respondents felt dissatisfied with their income and it was not related to gender and type of responsibility. Surprisingly, owners and non-owners were both unsatisfied with their income. This result differs from the study examined Lebanese pharmacists’ satisfaction, in which most of interviewed pharmacists were financially satisfied and pharmacy owners were “the most financially satisfied individuals in the study” (14).

There are two types of pharmacists working at Iran private-sector pharmacies: 1. The non-owners, who are employed by the owners to provide pharmaceutical services, mostly dispensing prescriptions, 2. The owners of pharmacies who pay the employed pharmacists and are responsible for business in pharmacies. Non-owners are paid a fixed amount per hour and the salary does not depend on the quantity or quality of services. As extra, the number of dispensed items and the time spent on patient counseling and mark-up, do not make any difference to employed pharmacists’ salary. Subsequently, employed pharmacists just focus on dispensing activity and their main concerns are medication errors. Furthermore, there are no incentives for providing further services like patient counseling. In contrast, the owners of pharmacies are always concerned about the income of pharmacies and they are always willing to increase sales. As the results reflect, owners significantly experience longer work hours and more workload pressures than non-owners.

In an article published in 2009, authors analyzed various models of remuneration in different countries (15). In Iran, there is mark-up system and also pharmacies receive part of their income from fixed dispensing fees paid by patients for dispensing each item. As discussed in different articles, mark-up method encourages pharmacists to have product-oriented approaches (15, 16). Thus, those who are concerned about the quality of their work life among pharmacists in Iran should endeavor to develop suggested models (15) to pay pharmacists for providing professional services beyond supplying medicines. This approach will provide incentives for pharmacists to improve the professional satisfaction as well as the income satisfaction since they feel rewarded for their professional performances.

Based on our results, younger pharmacists were significantly less satisfied with their incomes compared to older pharmacists. Moreover, the young practitioners are more likely to experience dissatisfaction since their ideal expectations differ from reality (17). Consequently, as income is one of the main predictors of job satisfaction, young pharmacists in Iran are more likely to experience less job contentment and it might endanger their quality of professional life.

It was also suggested that the respondents› job satisfaction and quality of life were not significantly different between the sexes. Results about sex-related satisfaction are controversial and some studies reported men are less satisfied compared to women (6).

Almost half of the respondents reported not being relaxed while working at pharmacy. Previous studies showed that the work overload, the short staffed and being anxious about the medication errors, are major factors which cause pharmacists’ job stress (3, 10). Sources of stress among dispensing pharmacists in Iran should be studied in the future according to the modified models (18). 

## Conclusions

Taken as a whole, the general health and quality of life among the respondents are at a satisfactory level. However, their work-related satisfaction was not high enough and most interviewed pharmacists reported low income as the main reason of job dissatisfaction. 

The present study was conducted in small sample size in Tehran. Therefore, stated results need further research to be conclusive. In addition, this study was done among dispensing pharmacists. Hence, conducting a survey among all pharmacy sectors should be arranged in the future. 
